# Is the Addition of CO_2_ Laser to β3-Adrenoceptor Agonist Mirabegron Effective in the Management of Overactive Bladder? Results of a Randomized Controlled Trial

**DOI:** 10.3390/medicina61071198

**Published:** 2025-06-30

**Authors:** Konstantinos Kypriotis, Anastasia Prodromidou, Stavros Athanasiou, Dimitrios Zacharakis, Nikolaos Kathopoulis, Athanasios Douligeris, Veatriki Athanasiou, Lina Michala, Themos Grigoriadis

**Affiliations:** 1 Urogynecology Unit, 1st Department of Obstetrics and Gynaecology, “Alexandra” General Hospital, National and Kapodistrian University of Athens, 11528 Athens, Greece; kypriotisk@gmail.com (K.K.); aprodromidou@med.uoa.gr (A.P.); athanasio@med.uoa.gr (S.A.); dimzac@hotmail.com (D.Z.); nickatho@gmail.com (N.K.); linamichala@med.uoa.gr (L.M.); 2 Medicine, Brighton and Sussex Medical School, Brighton BN1 9PX, UK; beatrice.ath@gmail.com

**Keywords:** overactive bladder, mirabegron, CO_2_ laser therapy, sham laser, randomized controlled trial

## Abstract

*Background and Objectives*: This study aimed to assess whether the addition of fractional CO_2_ laser therapy to standard pharmacologic treatment with Mirabegron, a β3-adrenoceptor agonist, enhances the clinical outcomes in the management of overactive bladder syndrome (OAB) in postmenopausal women. *Materials and Methods*: Τhis was a prospective, randomized, double-blind, sham-controlled trial including 50 postmenopausal women with moderate-to-severe OAB symptoms. Participants were randomized (1:1) to receive mirabegron 50 mg daily in combination with either active fractional CO_2_ laser therapy (Group A) or sham laser treatment (Group B). Both groups underwent three monthly sessions of vaginal laser treatment and were followed for a total of four months. Clinical assessments were performed at baseline and monthly visits (T0–T3), using validated instruments including the Overactive Bladder Questionnaire (OAB-q), King’s Health Questionnaire (KHQ), Urinary Distress Inventory (UDI-6), Pelvic Floor Impact Questionnaire (PFIQ-7), Patient Global Impression of Improvement (PGI-I), and 3-day voiding diaries. The trial was registered at ClinicalTrials.gov (Identifier: NCT03846895). *Results*: Significant symptom improvement was observed within both groups over time, with reductions in urinary frequency, urgency, nocturia, and incontinence episodes, as well as improvements in quality-of-life scores. However, intergroup comparisons revealed no statistically significant differences in any primary or secondary outcomes. Both treatment modalities demonstrated similar effectiveness across all measured parameters. *Conclusions*: In this randomized controlled trial, the adjunctive use of fractional CO_2_ laser therapy did not offer additional clinical benefit beyond mirabegron monotherapy in the short-term management of OAB. These findings underscore the need for further investigation into tailored therapeutic strategies, particularly in populations with overlapping genitourinary syndrome of menopause or more refractory OAB symptoms.

## 1. Introduction

Overactive bladder (OAB), as defined by IUGA/ICS, is a condition marked by urinary urgency, nocturia, frequency, and may occur with or without urgency urinary incontinence, without the presence of urinary tract infection or other clear pathology [1,2]. The prevalence of OAB increases with age, particularly affecting postmenopausal women, with an estimated incidence of approximately 12–22% among the adult population [3,4]. OAB can significantly impair quality of life, leading to social embarrassment, anxiety, and depression [5,6]. The management of OAB typically involves lifestyle changes, pelvic floor muscle training, pharmacotherapy with antimuscarinics or β3-adrenoceptor agonists, or a combination of them [7]. Mirabegron is a selective β3-adrenoceptor agonist that is widely used in the management of overactive bladder (OAB). Mirabegron has been reported to be equally effective to antimuscarinics in increasing the bladder capacity and reducing the urgency, frequency, and incontinence episodes without the anticholinergic adverse effects [8]. Its safety and efficacy have been confirmed in both short- and long-term clinical trials, making it a reasonable pharmacological treatment of OAB [8].

In refractory cases, more invasive options such as sacral neuromodulation, botulinum toxin injections, or surgery have been proposed [9]. Additionally, local estrogen therapy has also been utilized in postmenopausal women based on the relationship between OAB symptoms and genitourinary syndrome of menopause (GSM) [7,10]. The use of the combination of vaginal estrogen therapy and anticholinergics has also been investigated for the relief of OAB symptoms in women with GSM [11]. However, the variable therapeutic efficacy of the abovementioned treatments and concerns regarding the risk of breast cancer of hormonal treatments highlight the need for novel therapeutic approaches.

The application of laser therapy in medicine has grown exponentially in recent years. CO_2_ lasers are being widely used for cutting, ablating, and resurfacing tissues. For urogynecological indications, fractional CO_2_ laser therapy has shown potential as a promising alternative therapeutic option [12]. The underlying pathophysiological mechanism is believed to involve the stimulation of neocollagenesis, angiogenesis, and restoration of the extracellular matrix, resulting in the rejuvenation of the vaginal mucosa and the improvement of the integrity and function of the urogenital tissue [13]. A wide variety of studies have recorded beneficial outcomes of laser CO_2_ in relief of GSM symptoms as an alternative option to topical vaginal estrogen therapy [14]. Interestingly, laser therapy has been associated with a significant reduction in both prevalence and severity of lower urinary tract symptoms in patients with GSM [15]. However, despite the encouraging outcomes in the subpopulation of GSM patients, studies on the efficacy of fractional CO_2_ laser in the management of OAB symptoms are still limited.

Based on the efficacy of mirabegron and the emerging data on fractional CO_2_ laser therapy for GSM, the combination of them could potentially result in a synergistic effect, particularly in the subset of postmenopausal women. However, evidence on such a combination is lacking. The objective of this study is to assess the potential efficacy of the combined treatment with CO_2_ laser and β3-adrenoreceptor agonist compared to monotherapy with β3 adrenoreceptor agonist for managing OAB in postmenopausal women.

## 2. Materials and Methods

### 2.1. Study Design

This was a double-blind randomized controlled trial on postmenopausal women with symptoms of overactive bladder according to the International Consultation on Incontinence Questionnaire for the evaluation of the Female Lower Urinary Tract (ICIQ-FLUTS) and who were randomized to receive vaginal treatment with fractional CO_2_ laser or sham control from 04, 2018.

The inclusion criteria were as follows: menopausal status defined as ≥12 months of amenorrhea or FSH ≥ 30–40 mIU/mL after total hysterectomy; symptoms of OAB including ≥3 months of urgency symptoms, with or without urinary incontinence, and ≥8 episodes/24 h; at least 3 episodes of urgent urination (3rd–4th grade) under Patient Perception intensity of Urgency Scale (PPiUS) during a three-day urination calendar, with or without urinary incontinence. Exclusion criteria included women with genital prolapse > stage II according to the Pelvic Organ Prolapse Quantification system (POP-Q), voiding residual volume > 200 mL, bladder obstruction, vaginal estrogen use during the last month, previous chronic use of medication for urinary incontinence, use of medication for psychiatric disorders, urinary or genital infection, kidney or liver disease, congestive cardiac failure or rare disorders, unregulated arterial hypertension, neurological diseases and diabetic neuropathy, presence of myasthenia, malignancy, and previous pelvic radio-chemotherapy.

This study was performed in an outpatient urogynecological clinic and approved by the Institutional Review Board of “Alexandra” General Hospital of Athens (Protocol number: 303/16-04-2018). The study protocol was registered at ClinicalTrials.gov (Identifier: NCT03846895) on 20 February 2019, prior to the first patient enrollment. The local ethics committee approved the clinical protocol in adherence with the Declaration of Helsinki and all patients signed an informed consent. The study protocol was designed in accordance with the Standard Protocol Items: Recommendations for Interventional Trials (SPIRIT) 2013 guidelines [16], and the trial was conducted and reported according to the Consolidated Standards of Reporting Trials CONSORT 2010 guidelines [17]. 

### 2.2. Randomization

Eligible patients were randomized in a ratio of 1:1 prior to the 1st treatment, using a computer-generated randomization scheme, to receive active laser therapy or placebo, once monthly for three months.

### 2.3. Intervention

All participants received treatment with mirabegron 50mg and they were equally divided into active or sham laser therapy groups. Fractional CO_2_ laser (SmartXide2 V2LR, Monalisa Touch, DEKA, Florence, Italy) was applied at monthly intervals for both groups for a total of 3 treatments.

The standard settings were applied for the laser treatment (power: 40 watts, dwell time: 1000 μs, DOT spacing at 1000 μm, SmartStack 3 on D-pulse emission mode), delivering fluence of 5.37 J/cm^2^. The sham treatment was performed at a power of 0.5 watts, dwell time 1000 μs, DOT spacing at 1000 μm, and smart-pulse emission mode delivering no energy (fluence, 0 J/cm^2^).

### 2.4. Blinding

The investigator, study site personnel, and patients were blinded to treatment. An unblinded nurse was responsible for programming the laser parameters, before the doctor and patient entered the examination room. The screen of the laser system was covered to ensure proper blinding. Although in the placebo group the power of 0.5 watts and smart pulse emission mode would not deliver energy to the vaginal tissue, the identical laser sound and treatment technique as in the active group covered the placebo procedure.

### 2.5. Outcomes

This study consisted of four monthly clinical visits (T0, T1, T2, T3). Patient demographics and other baseline characteristics were collected at the start of the run-in period just before the first treatment (T0). Patients completed the following questionnaires: the Overactive Bladder Questionnaire (OAB-q); King’s Health Questionnaire (KHQ); Urinary Distress Inventory (UDI-6); and Pelvic Floor Impact Questionnaire (PFIQ-7) at every visit and a three-day micturition diary a week before every visit. The questionnaires were completed before first (T0), second (T1), and third (T2) treatments as well as at follow-up one month after the last treatment (T3). In addition, the Patient Global Impression of Improvement questionnaire (PGI-I) was added in the last visit. Patients were advised to record twice daily their blood pressure and pulse rate, a week before the randomization and at every visit.

The primary outcomes were evaluated as a change in the severity of symptoms as assessed by the score of the OAB-q questionnaire that consists of an 8-item symptom bother scale section and a 25-item health-related quality of life scale section. Additionally, a 3-day voiding diary was used for monitoring the frequency of micturition, urgency, and urinary incontinence.

The secondary outcomes was assessed by the difference in scores of the following validated questionnaires: KHQ, UDI-6, PFIQ, and PGI-I.

### 2.6. Sample Size Calculation

Power analysis methodology represents a design, with two levels of the between-subject factors of two study groups and four levels of the within-subjects factor of time. A repeated measures ANOVA power analysis was conducted. The effect size for this calculation used the ratio of the standard deviation of the effects for a particular factor or interaction and the standard deviation of within-subject effects. The power analysis was conducted for a single, two-group between-subjects factor, and a single within-subjects factor assessed over four time points. For this design, 50 participants (25 per group) achieve a power of 0.80 for the within-subjects main effect at an effect size of 0.25; a power of 0.80 for the between-subjects main effect at an effect size of 0.32; and a power of 0.80 for the interaction effect at an effect size of 0.25.

### 2.7. Statistical Analysis

The analyses were conducted using SPSS statistical software (version 22.0). Comparison of data between the compared groups in both phases was based on the intention-to-treat principle. The intention-to-treat participants were all randomized women independently of whether they received therapies (active or placebo), or they dropped out from this study, or adhered to the study’s protocol.

Quantitative variables were expressed as mean (Standard Deviation) or as median (interquartile range). The normality assumption was evaluated using Kolmogorov–Smirnov test. Student’s *t*-tests were computed for the comparison of a continuous variable between two groups when the distribution is normal and the Mann–Whitney test when the distribution is not normal. Paired *t*-tests and Wilcoxon signed rank tests were used for pre- and post-comparisons. For the comparison of proportions between the two study groups, chi-square and Fisher’s exact tests were used. Repeated measurements analysis of variance (ANOVA) was performed to evaluate the changes observed between control and intervention group over the follow up period. Additionally, three sensitivity analyses were performed: per-protocol analysis, robustness of trial accounting for missing data, and adjusting for baseline covariates. 

The OAB-q symptom bother scale was the pre-specified primary endpoint. Secondary endpoints included validated questionnaires (KHQ, UDI-6, PFIQ-7) and diary variables. Repeated measurements analysis of variance was conducted after logarithmic transformation of the parameters. Bonferroni correction was used in the case of multiple testing in order to control for type I errors. Bonferroni correction was applied for within-subject repeated testing; however, family-wise error control was not applied across all secondary outcomes, which are considered exploratory.

Adjustment for confounding factors will be performed using mixed linear regression models that accounts for multiple measurements per individual obtained at different time points. All reported p values will be two-tailed and statistical significance is set at *p* < 0.05.

## 3. Results

**Figure 1:**
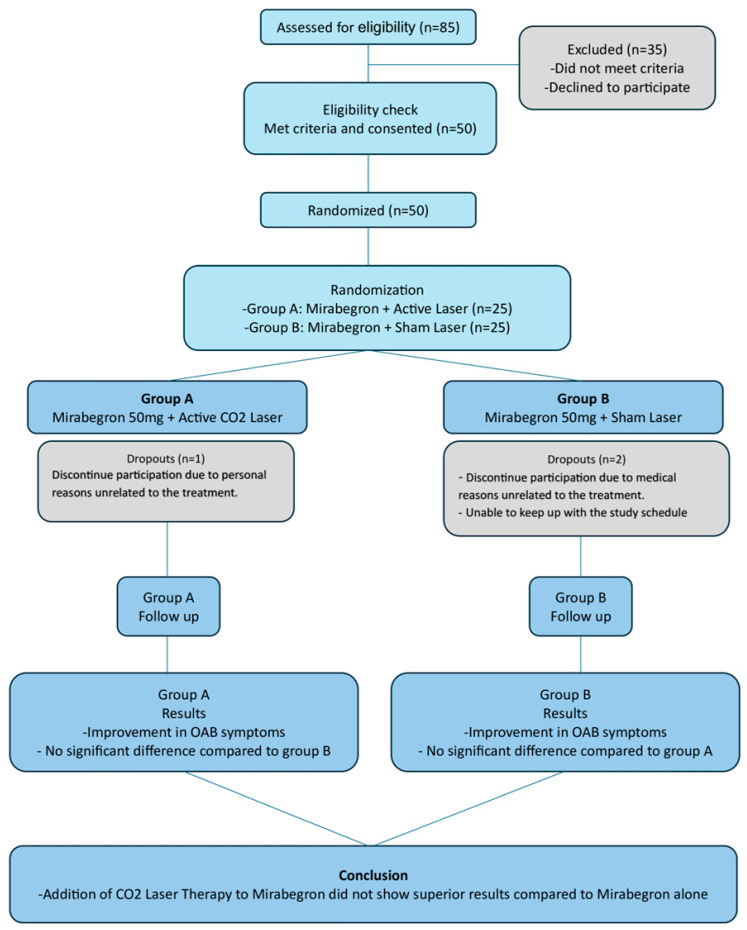
CONSORT flow diagram of participant progression through the phases of the trial.

Of the 85 women assessed, 50 were recruited and randomized in two equal-sized group of 25 women each (active/co-therapy versus sham/monotherapy groups). A CONSORT diagram illustrating the number of participants assessed for eligibility, randomized, treated, and analyzed is presented in Figure 1.

The mean age of patients was 62.3 years (SD = 6.4 years). Patients’ characteristics are presented in Table 1, by group. No differences were observed among the two groups in patients’ characteristics. All patients completed the treatment and the follow-up 1 month after the last session.

Changes in urinary outcomes throughout the follow-up period, by group, are presented in Table 2. Despite the fact that all urinary outcomes except mean urinary volume were significantly improved over time, in both groups, the intergroup comparison revealed no difference in each session separately.

Daily frequency significantly decreased from T0 to T3 in both the active (*p* < 0.001) and sham group (*p* < 0.001). This was also detected for both groups in the case of T0-T3 comparison of daily urgency (*p* < 0.001), nocturia (*p* < 0.001), and daily incontinence episodes (*p* = 0.0.002 and *p* = 0.019, for active and sham group, respectively). Mean total volume from T0 to follow-up was significantly reduced in both groups (*p* = 0.005). However, despite the fact that the mean urinary volume at each urination was increased in both groups from T0 to T3, this was only significant in the case of the sham group (*p* = 0.022).

Changes in UDI6 and UIQ-7 scores throughout the follow-up period, by group, are presented in Table 3. The two groups did not differ in each session separately. Both scales changed significantly over time, in both groups, in a similar way. More specifically, after Bonferroni correction, from T0 to T3, there was a significant decrease in the UDI6 score in both the active (*p* < 0.001) and sham group (*p* = 0.001). Overall, from T0 to T3, there was a significant decrease in the UIQ-7 score in both the active (*p* < 0.001) and sham group (*p* < 0.001).

Changes in KHQ scale over the follow-up period, separated by group, are detailed in Table 4. There were no significant differences between the two groups in comparison of each session separately, except for the general health perception score, which was significantly decreased in sham group compared to the active group at follow-up (T3), (*p* = 0.01). Most domains including incontinence impact, role, social and physical limitations, emotions, sleep/energy, severity measures, and symptom severity, changed significantly over time, with both groups demonstrating better outcomes without marked differences between them. However, general health perception and personal relationships score presented significant changes from T0 to T3 only in the case of the sham monotherapy group (*p* = 0.001 and *p* = 0.028, respectively).

Changes in OAB-q scale throughout the follow-up period, by group, are presented in Table 5. No statistically significant differences were observed among the two groups in each session separately. All scales changed significantly over time, presenting better outcome for both groups, in a similar way. Overall, from T0 to T3, there was a significant increase in total OAB-q score and of all individual domains (symptom severity, coping, concern, sleep, social interactions) in both groups.

The incidence of adverse events was low across both groups, and no serious adverse events were recorded. Vaginal discomfort was the most commonly reported issue, occurring in five women in the laser + mirabegron group and three women in the sham + mirabegron group. Spotting or mild vaginal bleeding was reported by one participant in each group. No urinary tract infections were recorded in the laser group, whereas one case was noted in the sham group and resolved with oral antibiotics. Transient elevations in blood pressure were observed in two participants in the laser group and three in the sham group, without requiring intervention. Overall, both treatment regimens were well tolerated with minimal and self-limiting side effects.

## 4. Discussion

The management of overactive bladder in women in menopause often poses a significant clinical challenge. This study aimed to assess the effectiveness of a combined therapy using a b3 adrenoreceptor agonist and fractional CO_2_ laser as a treatment modality for OAB symptoms in this population. Our findings indicate that while there were notable improvements in urinary outcomes within each group over time, no statistically significant differences were detected between the active (laser) group and the sham control group.

Mirabegron, a β3-adrenoceptor agonist, is crucial in the management of OAB. Its mechanism of action involves the selective activation of β3-adrenoceptors in the detrusor muscle of the bladder, which leads to relaxation of the bladder muscle during the storage phase and consequently increases bladder capacity. Unlike antimuscarinic agents that are traditionally used for OAB, mirabegron could be a valuable option in reducing urinary frequency and incontinence episodes without causing the typical side effects such as dry mouth and constipation. Its efficacy and safety profile have been confirmed by numerous clinical trials, making it an important therapeutic tool in improving the quality of life in OAB patients. According to a phase III clinical trial, administration of either 50mg or 100mg of mirabegron resulted in improved OAB symptoms compared to placebo, with a significant reduction in episodes of urinary incontinence and an increase in voided volume per micturition [18]. This was also observed in the post-hoc analysis of the same study population in subgroups according to patients in prior antimuscarinic therapy and those who were antimuscarinic naive [19]. Additionally, further long-term studies have demonstrated the sustained efficacy and safety of mirabegron over 12 months, affirming its role as a viable long-term treatment option for patients with OAB [20]. Finally, compared to antimuscarinics, a recent meta-analysis proved that despite the comparable therapeutic efficacy, the number of adverse events in the mirabegron group was significantly decreased [8].

Although fractional CO_2_ laser has shown promising outcomes in the management of GSM, its application in OAB management is less well understood. The effects of CO_2_ laser are attributed to the induced tissue regeneration that leads to improved vaginal health and function [21]. The thermal stimulation from the CO_2_ laser is thought to promote neocollagenesis and microvascularization, which could theoretically enhance the structural support of the bladder and urethra, thereby reducing symptoms of urgency and frequency [21,22]. More specifically, CO_2_ laser has been found to induce its effect deeper in the vaginal wall layers and could potentially involve the symptoms associated with the bladder and urethra by enhancing proliferation of extracellular matrix and bladder blood supply [22]. However, it is unclear whether these tissue-level changes may translate into clinical symptom relief for OAB.

We observed significant improvement in symptomatic relief of OAB symptoms with the use of laser CO_2_ during the follow-up interval as assessed by the 3-day voiding diary and patients self-reported questionnaires. Outcomes on the efficacy of laser CO_2_ are consistent with the preliminary outcomes by Perino et al. who conducted a single-arm prospective study on 30 post-menopausal patients with OAB symptoms who received three laser CO_2_ sessions. More specifically, the authors observed significantly improved micturition diaries and decreased urge episodes after treatment (*p* < 0.0001) [23].

Based on the abovementioned encouraging observations of the CO_2_ laser therapy, this study aimed to explore the possible synergistic effect of combining two different treatment modalities. To the best of our knowledge, this is the first study to assess the efficacy of the co-therapy using a b3 adrenoreceptor agonist plus CO_2_ laser compared to monotherapy with mirabegron alone. In the randomized controlled trial by Aguiar et al., postmenopausal women with GSM-related urinary symptoms received monotherapy with either fractional CO_2_ laser, vaginal promestriene, or vaginal lubricant [24]. Despite the significant decrease in nocturia in the CO_2_ laser group before and after treatment, the superiority of the laser was only evident in comparison to the ICIQ-OAB score with the lubricant group [24]. The use of non-ablative vaginal erbium, YAG laser (VEL), has also been studied. In particular, Okui et al. recorded a comparable therapeutic effect of VEL and pharmacotherapy with either the anticholinergic fesoterodine or mirabegron in the management of OAB symptoms [25]. However, compared to pharmacotherapy, the VEL group also presented a favorable safety profile with no reported adverse events [25]. Treatment of GSM-related OAB symptoms with the use of VEL was also assessed by the randomized sham-controlled trial by Chiengthong et al. [26]. Compared to the sham control group, the VEL group demonstrated significantly improved changes in total OAB score (*p* = 0.015) as well as when nocturia and urgency were separately analyzed (*p* = 0.004 and *p* = 0.008, respectively) [26].

The lack of superiority in the co-therapy group could be attributed to several factors. First of all, the mirabegron itself seems to have a high efficacy in relieving OAB symptoms [8,27]. Its proven efficacy might overshadow any potential additional benefit provided by the CO_2_ laser therapy. This high baseline efficacy of mirabegron could make it challenging to detect any incremental improvements from the co-therapy with CO_2_ laser. Ιt is also plausible that longer follow-up could reveal delayed or more sustained benefits of fractional CO_2_ laser therapy. Previous studies have suggested that tissue remodeling and collagen synthesis, stimulated by laser energy, may progress over several months [21]. Therefore, the 1-month post-treatment follow-up might underestimate the full therapeutic potential, particularly the long-term symptom control and durability of response. Another significant reason is that the study population included postmenopausal women with OAB but did not specifically focus on the subgroup with GSM. Since earlier studies have proved that CO_2_ laser therapy is particularly effective in managing GSM-related symptoms [15], the inclusion of women without primary GSM symptoms might have diluted the potential benefits of the laser therapy. A more targeted population of OAB and GSM symptoms might have shown a clearer distinction between the co-therapy and monotherapy groups. In addition to this, patients of advanced age and more years of menopause could potentially represent more resistant cases. Future studies and analyses should stratify participants based on the presence and severity of GSM, as CO_2_ laser could be particularly effective in this subgroup.

From a health economics perspective, evaluating the cost-effectiveness of CO_2_ laser as an adjunctive therapy is essential. Laser treatments typically involve capital equipment and procedural costs, which may not be affordable for all healthcare systems. Future studies should incorporate formal cost-benefit analyses to better inform clinical and policy decisions.

One of the main strengths of the present study is its randomized, double-blind design. Additionally, the comprehensive assessment of outcomes using multiple validated questionnaires and the 3-day voiding diary provides a thorough evaluation of the treatment’s impact on patients’ symptoms and quality of life.

However, there are also limitations to be considered. The sample size, while calculated to achieve adequate power for detecting differences, may still be insufficient to observe small but clinically meaningful effects. In addition to this, given the small effect sizes and non-significant results, a post-hoc power analysis was not conducted, as it would offer limited additional value. Future multicenter studies with larger cohorts may provide more definitive evidence on the additive value of CO_2_ laser. Additionally, the duration of follow-up may not have been long enough to capture the long-term effects of laser therapy on OAB symptomatology. In particular, some therapeutic effects, especially those related to tissue regeneration and long-term symptom relief from CO_2_ laser therapy, might require a longer time to become apparent. A longer follow-up period could potentially reveal differences in the durability and sustained efficacy of the treatments that were not evident within the shorter timeframe. In addition to this, the study protocol included three monthly sessions at monthly intervals of fractional CO_2_ laser therapy. This is consistent with several previous trials investigating laser treatment for OAB and genitourinary syndrome of menopause (GSM) symptoms [20,23]. This might not have been sufficient to induce a noticeable therapeutic effect in managing OAB symptoms. Emerging data—particularly from studies in GSM—suggest a potential dose–response relationship, with improved outcomes observed after four or even five treatment sessions [13]. While a definitive dose–response curve has yet to be established, the incremental benefit of additional sessions warrants further investigation, particularly in patients with more severe symptoms or in whom monotherapy with mirabegron is inadequate. Given the favorable safety profile of fractional CO_2_ laser therapy, exploring a higher number of sessions (up to five) or adjusting the treatment frequency in future randomized trials appears ethically feasible, especially when patient selection is guided by symptom severity and/or baseline GSM status. Future studies should aim to determine the optimal treatment frequency and assess whether extended laser therapy results in more durable or pronounced improvements. Another important limitation is the absence of a validated baseline assessment of GSM, such as the Vaginal Health Index (VHI) or Vaginal Maturation Index (VMI). Given that CO_2_ laser therapy has demonstrated greater efficacy in patients with symptomatic GSM, the lack of GSM stratification may have interfered with the ability to detect a differential response. While post-hoc subgroup analysis was considered, our sample size was insufficient to support statistically meaningful stratification. Future studies should incorporate objective GSM assessments to enable targeted evaluation of treatment effects in relevant subpopulations. Finally, a further study could explore extended regimens, particularly when CO_2_ laser is administered as monotherapy. To that end, further research should consider a 2 × 2 factorial design to distinguish between the independent and synergistic effects of CO_2_ laser and mirabegron, including the use of a mirabegron-naïve cohort for initial assessment.

It would also be beneficial to explore the mechanisms by which laser therapy may influence bladder function and to determine whether certain subpopulations of women with OAB may respond more favorably to this treatment. Moreover, comparative studies with other non-pharmacological treatments including other laser types or platelet rich plasma could help position fractional CO_2_ laser therapy within the broader context of OAB management options. Finally, this study lacks evaluation of the cost-effectiveness of the examined treatments.

## 5. Conclusions

This randomized, double-blind study assessed the potential of CO_2_ laser as an adjuvant therapy alongside β3-adrenoreceptor agonists for managing OAB in postmenopausal women. The results indicate significant symptomatic improvement within both the treatment and control groups; however, no discernible differences between the groups were observed. This implies that while the laser treatment shows promise, its clinical efficacy for OAB symptoms still remains unclear. Future studies should consider an extended follow-up and stratification by GSM severity, to better evaluate the role of CO_2_ laser in OAB management and to explore its synergistic potential effect with OAB pharmacotherapies.

## Figures and Tables

**Table 1:** Main patient characteristics by group.

		Group		*p*
		Active (Ν = 25; 50%)	Sham Ν = 25; 50%)	
		N (%)	N (%)	
Age, mean (SD)		61.5 (6.6)	63.1 (6.3)	0.372 ^+^
NVD, median (IQR)		2 (1–2)	2 (1–2)	0.417 ^++^
BMI, mean (SD)		28.6 (4.4)	28.1 (4.8)	0.711 ^+^
BMI				
	Normal	5 (20)	5 (20)	0.599 ^‡^
	Overweight	12 (48)	15 (60)	
	Obese	8 (32)	5 (20)	
Age at menopause, mean (SD)		50.6 (4.2)	49.8 (3.7)	0.457 ^+^
Years of OAB symptoms, median (IQR)		4 (3–7)	4 (3–5)	0.550 ^++^
History of abdominal surgery		12 (48)	12 (48)	1.000 ^‡^
Hypertension		6 (24)	3 (12)	0.463 ^‡‡^
Condition after CO_2_ laser treatment	Very much better	4 (16)	3 (12.5)	0.796 ^‡‡^
	Much better	12 (48)	10 (41.7)	
	A little better	9 (36)	11 (45.8)	

^+^ Student’s *t*-test; ^++^ Mann–Whitney test; ^‡^ Pearson’s x^2^ test; ^‡‡^ Fisher’s exact test.

**Table 2:** Changes in urinary outcomes based on 3-day voiding diary throughout the follow-up period by group.

		Session 0	Session 1	Session 2	Session 3	Change		
	Group	Mean (SD)	Mean (SD)	Mean (SD)	Mean (SD)	Mean (SD)	*p* ^2^	*p* ^3^
Frequency/Day	A	12.2 (3)	10.4 (2.3)	9.1 (1.4)	8.5 (1.6)	−3.7 (2)	<0.001	0.639
	Β	11.6 (3.2)	10.3 (2.9)	8.7 (2.4)	8.4 (2.1)	−3.2 (2.3)	<0.001	
	*p* ^1^	0.359	0.771	0.292	0.705			
Urgency/Day	A	5.31 (3.62)	2.23 (1.75)	1.58 (2.02)	1.11 (1.74)	−4.2 (3.49)	<0.001	0.091
	Β	4.05 (3.24)	2.35 (1.44)	1.61 (1.37)	0.93 (1.23)	−3.12 (2.68)	<0.001	
	*p* ^1^	0.900	0.840	0.573	0.869			
Nocturia/day	A	1.91 (1.03)	1.54 (0.78)	1.25 (0.62)	1.11 (0.77)	−0.8 (0.71)	<0.001	0.316
	Β	1.87 (1.02)	1.35 (0.72)	1.04 (0.66)	1.17 (0.67)	−0.7 (0.85)	<0.001	
	*p* ^1^	0.961	0.489	0.276	0.595			
Mean Total Volume	A	2027.6 (681.1)	1953.4 (607.3)	1770.5 (631.4)	1723.1 (687.6)	−304.4 (577.1)	0.005	0.374
	Β	2085.7 (597.4)	1927.8 (476.5)	1772.8 (549.8)	1843.3 (558.6)	−242.5 (503.1)	0.005	
	*p* ^1^	0.643	0.911	0.794	0.304			
Mean Urinary Volume	A	177.4 (69.2)	195 (71.8)	197.2 (74.1)	205.2 (78.8)	27.8 (57.3)	0.100	0.455
	Β	191.7 (61.8)	194.6 (59.3)	212.2 (64.3)	230.9 (90.9)	39.2 (84.3)	0.022	
	*p* ^1^	0.389	0.873	0.365	0.255			
Incontinence Episodes/Day	A	2.19 (4.12)	0.68 (1.54)	0.43 (1.41)	0.4 (1.43)	−1.79 (3.78)	0.002	0.431
	Β	1.44 (2.33)	0.76 (1.56)	0.66 (1.69)	0.62 (1.69)	−0.82 (1.34)	0.019	
	*p* ^1^	0.737	0.770	0.460	0.588			

Group A: active/co-therapy group, Group B: sham group, *p*^1^-value for group comparison at each session, *p*^2^-value for comparison among T0 and T3 (follow-up) *p*^3^-repeated measures ANOVA. Effects reported include differences between the groups in the degree of change over the follow-up period. Note: analysis was conducted after logarithmic transformation of the variables.

**Table 3:** Changes in UDI6 and UIQ-7 scores throughout the follow-up period, by group.

		Session 0	Session 1	Session 2	Session 3	Change		
	Group	Mean (SD)	Mean (SD)	Mean (SD)	Mean (SD)	Mean (SD)	*p* ^2^	*p* ^3^
UDI6 score	A	49.2 (18.1)	36 (19.2)	29.5 (18.9)	23.7 (15.3)	−25.5 (21.1)	<0.001	0.896
	Β	52.2 (23.1)	39.2 (17.1)	28.5 (14.9)	25.7 (20.6)	−26.5 (27.6)	0.001	
	*p* ^1^	0.839	0.384	0.520	0.900			
UIQ-7 score	A	53.1 (21.9)	38.7 (24.2)	28.8 (22.1)	21.5 (19.3)	−31.6 (24)	<0.001	0.611
	Β	56.6 (24)	32.6 (22.6)	22.3 (20.6)	20 (20.3)	−36.6 (25)	<0.001	
	*p* ^1^	0.760	0.464	0.371	0.543			

Group A: active/co-therapy group; Group B: sham group; *p*^1^-value for group comparison; *p*^2^-value for time comparison; *p*^3^-repeated measures ANOVA. Effects reported include differences between the groups in the degree of change over the follow-up period. Note: analysis was conducted after logarithmic transformation of the variables.

**Table 4:** Changes in KHQ scale throughout the follow-up period, by group.

		Session 0	Session 1	Session 2	Session 3	Change		
	Group	Mean (SD)	Mean (SD)	Mean (SD)	Mean (SD)	Mean (SD)	*p* ^2^	*p* ^3^
General health perception	A	31 (24.2)	27 (19)	25 (16.1)	25 (17.7)	−6 (23.1)	0.969	0.103
	Β	27 (20.3)	23 (22.7)	20 (19.1)	14 (17.8)	−13 (21.8)	0.001	
	*p* ^1^	0.674	0.275	0.170	0.010			
Incontinence impact	A	77.3 (20.9)	56 (23)	46.7 (31.9)	37.3 (24.2)	−40 (30.4)	0.001	0.674
	Β	73.3 (23.6)	50.7 (17)	38.7 (26.7)	32 (24.5)	−41.4 (35.1)	<0.001	
	*p* ^1^	0.466	0.486	0.740	0.336			
Role limitations score	A	58 (25.5)	37.3 (29.4)	30 (24.1)	28 (27.1)	−30 (36.3)	0.001	0.091
	Β	55.3 (21.9)	31.3 (18.2)	22.7 (19.8)	18.1 (26.0)	−37.2 (30.5)	<0.001	
	*p* ^1^	0.624	0.470	0.564	0.166			
Physical limitations score	A	56.7 (26.8)	46.7 (34)	37.3 (30.5)	32 (29.2)	−24.7 (33.7)	0.002	0.919
	Β	56 (34)	34.7 (22)	25.3 (19.3)	23.3 (26.4)	−32.7 (36.2)	0.001	
	*p* ^1^	0.433	0.682	0.645	0.382			
Social limitations score	A	51.8 (27.6)	36.2 (24.8)	27.6 (23.5)	20.4 (14.1)	−31.3 (27.1)	<0.001	0.951
	Β	40.2 (26)	27.3 (20.7)	19.1 (15.1)	16.9 (15)	−23.3 (26.7)	<0.001	
	*p* ^1^	0.158	0.190	0.410	0.259			
Personal Relationships score	A	39.3 (41.7)	22.6 (27.4)	8.8 (14.6)	8.9 (13.9)	−30.4 (35.2)	0.366	0.506
	Β	23.5 (33.9)	17.9 (31)	3.3 (9.3)	6 (18)	−17.6 (27.3)	0.028	
	*p* ^1^	0.760	0.349	0.150	0.185			
Emotions score	A	48 (31.9)	32.4 (25)	25.8 (21.2)	21.3 (21)	−26.7 (32.7)	0.002	0.589
	Β	46.2 (34.6)	32.9 (27.7)	16.4 (17.9)	16.9 (20.8)	−29.3 (32.2)	<0.001	
	*p* ^1^	0.537	0.947	0.230	0.384			
Sleep/Energy score	A	43.3 (19.2)	32 (18.6)	28.7 (23.8)	20.7 (21.7)	−22.7 (21.5)	<0.001	0.547
	Β	40.7 (28.5)	34.7 (28.8)	22.7 (26.7)	20.7 (25.1)	−20 (22.6)	0.003	
	*p* ^1^	0.108	0.748	0.306	0.816			
Severity measures score	A	44 (26.2)	34.7 (21.9)	30.7 (22.1)	27 (25.7)	−17 (19.5)	0.001	0.954
	Β	42 (22.6)	31.7 (20.1)	26 (17.6)	21 (19.3)	−21 (18.5)	<0.001	
	*p* ^1^	1.000	0.979	0.904	0.812			
Symptom severity scale (0-10)	A	7.4 (1.89)	5.88 (2.4)	5.08 (2.33)	3.84 (2.85)	−3.56 (2.38)	<0.001	0.789
	Β	6.76 (2.05)	5.2 (2.14)	4.6 (2.02)	3.56 (2.31)	−3.2 (2.4)	<0.001	
	*p* ^1^	0.235	0.450	0.493	0.997			

Group A: active/co-therapy group; Group B: sham group; *p*^1^-value for group comparison; *p*^2^-value for time comparison; *p*^3^-repeated measures ANOVA. Effects reported include differences between the groups in the degree of change over the follow-up period. Note: analysis was conducted after logarithmic transformation of the variables.

**Table 5:** Changes in OAB-q scale throughout the follow-up period, by group.

		Session 0	Session 1	Session 2	Session 3	Change		
	Group	Mean (SD)	Mean (SD)	Mean (SD)	Mean (SD)	Mean (SD)	*p* ^2^	*p* ^3^
Symptom severity	A	43 (18.9)	27.9 (12.9)	19.8 (12)	17.1 (12.2)	−25.9 (19.6)	<0.001	0.552
	Β	43.3 (18.3)	23.3 (14.1)	18.5 (10.1)	16.3 (11.3)	−27 (20.5)	<0.001	
	*p* ^1^	0.925	0.285	0.828	0.718			
Coping	A	57.5 (19)	73.5 (21.1)	79.8 (20.3)	82.8 (18.6)	25.3 (21.5)	0.001	0.928
	Β	61.5 (22.6)	76.7 (16.3)	83 (15.7)	86 (16.5)	24.5 (22.5)	0.001	
	*p* ^1^	0.747	0.434	0.447	0.527			
Concern	A	68.5 (17.7)	80.8 (17.6)	84.6 (14.4)	89.6 (12.6)	21.1 (18.6)	<0.001	0.776
	Β	71.4 (19.6)	83.1 (19)	88.7 (11.2)	90.9 (12.5)	19.4 (18.4)	0.001	
	*p* ^1^	0.633	0.421	0.307	0.884			
Sleep	A	67.5 (16.7)	79.5 (16.3)	84.5 (17)	87.4 (15)	19.8 (13.6)	0.003	0.654
	Β	69 (24.2)	84 (17.6)	87.5 (15.2)	87.7 (15)	18.7 (22.6)	0.001	
	*p* ^1^	0.802	0.511	0.531	0.972			
Social interaction	A	76.8 (19.6)	92 (14.6)	95.2 (8.6)	94.1 (11.4)	17.3 (22.4)	0.004	0.917
	Β	81.6 (23.5)	93.4 (14.6)	97.6 (4.8)	97.4 (5.8)	15.8 (22)	0.010	
	*p* ^1^	0.652	0.788	0.235	0.186			
Total OAB-q score	A	66.4 (15)	80.6 (16)	85.2 (14.4)	87.9 (13.8)	21.4 (16)	<0.001	0.864
	Β	71.1 (19.3)	83.3 (15.1)	88.4 (11.5)	90 (11.6)	18.9 (18)	<0.001	
	*p* ^1^	0.507	0.546	0.450	0.692			

Group A: active/co-therapy group; Group B: sham group; *p*^1^-value for group comparison; *p*^2^-value for time comparison; *p*^3^-repeated measures ANOVA. Effects reported include differences between the groups in the degree of change over the follow-up period. Note: analysis was conducted after logarithmic transformation of the variables.

## Data Availability

The data presented in this study are available on reasonable request from the corresponding author. The data are not publicly available due to privacy or ethical restrictions.

## Abbreviations

The following abbreviations are used in this manuscript:

OAB	Overactive Bladder
GSM	Genitourinary Syndrome of Menopause
ICIQ-FLUTS	International Consultation on Incontinence Questionnaire for the evaluation of the Female Lower Urinary Tract
PPiUS	Patient Perception intensity of Urgency Scale
OAB-q	Overactive Bladder Questionnaire
KHQ	King’s Health Questionnaire
UDI-6	Urinary Distress Inventory-6
PFIQ-7	Pelvic Floor Impact Questionnaire-7
PGI-I	Patient Global Impression of Improvement
ANOVA	Analysis of Variance
